# The triglyceride-glucose index is associated with the severity of hepatic steatosis and the presence of liver fibrosis in non-alcoholic fatty liver disease: a cross-sectional study in Chinese adults

**DOI:** 10.1186/s12944-020-01393-6

**Published:** 2020-10-07

**Authors:** Wen Guo, Jing Lu, Pei Qin, Xiaona Li, Wenfang Zhu, Juan Wu, Nianzhen Xu, Qun Zhang

**Affiliations:** Department of Health Promotion Center, the First Affiliated Hospital with Nanjing Medical University, 300 Guangzhou Road, Nanjing, 210029 China

**Keywords:** Triglyceride-glucose index, Hepatic steatosis, Liver fibrosis, Non-alcoholic fatty liver disease, Insulin resistance, HOMA-IR, Predictive value

## Abstract

**Background:**

The triglyceride-glucose index (TyG) is a reliable predictor of non-alcoholic fatty liver disease (NAFLD). Its association with the severity of hepatic steatosis and liver fibrosis in NAFLD is poorly understood. This study evaluated the relationship between these factors in NAFLD.

**Methods:**

A total of 4784 participants who underwent ultrasonography were enrolled. Anthropometric and biochemical measurements were assessed. Participants with NAFLD were diagnosed by ultrasound. The degree of hepatic steatosis and liver stiffness was evaluated with transient elastography.

**Results:**

The TyG index was significantly correlated with the severity of hepatic steatosis and the presence of liver fibrosis in patients with NAFLD. TyG quartile values correlated with increasing prevalence of NAFLD (Q1 30.9%, Q2 53.3%, Q3 71.7%, and Q4 86.4%, *P* < 0.001) and with the presence of liver fibrosis (Q1 13.5%, Q2 17.6%, Q3 18.8%, and Q4 26.1%, *P* < 0.001). The AUROC for the TyG index to predict NAFLD was 0.761, resulting in a cut-off value of 8.7. However, the AUC value of the TyG index was 0.589 for liver fibrosis, which was insufficient to predict this condition. The adjusted odds of having hepatic steatosis or liver fibrosis were more strongly associated with TyG values compared with HOMA-IR.

**Conclusion:**

The TyG index is positively related to the severity of hepatic steatosis and the presence of liver fibrosis in NAFLD. The index also performed better than HOMA-IR.

## Background

Non-alcoholic fatty liver disease (NAFLD) is a spectrum of diseases, including simple steatosis, non-alcoholic steatohepatitis (NASH), liver fibrosis and cirrhosis. In recent decades, NAFLD has increasingly become a health problem because of its growing incidence and its connection with a number of chronic diseases [1, 2]. Its presence is a risk factor for further liver injury, diabetes mellitus, cardiovascular disease (CVD), renal failure, and cancer [3–5]. Considering its seriousness, a thorough understanding of the pathogenesis, risk factors, and early interventions for NAFLD is essential. In addition, a simple and effective diagnostic tool for early identification of an individual’s risk of NAFLD is also necessary.

The pathophysiology of NAFLD is not well understood; however, the role of insulin resistance (IR) in the initiation and progression of NAFLD has been recognized by many scholars [6, 7]. The triglyceride-glucose (TyG) index derived from triglyceride and fasting blood glucose has recently been proven to be a reliable surrogate marker of IR. Hyperinsulinemic-euglycemic clamp test is the gold standard technique for measuring IR and has confirmed the relationship between the TyG index and IR [8, 9]. Evidence suggests that the TyG index is tied to metabolic diseases and CVD [10, 11]. The TyG index has been considered a reliable biomarker for predicting type 2 diabetes mellitus (T2DM) and metabolic syndrome (MS) [12, 13]. Several studies have even reported that the TyG index is a better predictor for coronary artery atherosclerosis and arterial stiffness when compared with HOMA-IR [14, 15]. Additionally, the correlation of the TyG index with NAFLD has drawn interest. Two studies, one cross-sectional and the other prospective, have both shown that the TyG index is a predictor of incident NAFLD, and both concluded that it may be a good diagnostic tool for NAFLD [16, 17]. However, little is known about the link between the TyG index and liver fibrosis or the severity of hepatic steatosis in NAFLD. Whether the TyG index is able to detect liver fibrosis risk in NAFLD has also not been investigated.

HOMA-IR most frequently relies on evaluating IR in clinical practice and has been demonstrated as an independent predictor of advanced fibrosis in NAFLD [18]. Salgado AL et al. found that HOMA-IR values above or equal to 2.0 or 2.5 could distinguish nondiabetic patients with NAFLD from a control group [19]. A cross-sectional study conducted in Korean adults showed that the TyG index predicted NAFLD better than HOMA-IR [20]. However, there are no data comparing the predictive power of the TyG index and HOMA-IR for NAFLD in Chinese adults.

Therefore, the aim of this study was to explore the association of the TyG index with the severity of hepatic steatosis and liver fibrosis in Chinese adults and compare the data with that of HOMA-IR. Another aim of this study was to explore the ability of the TyG index to identify individuals with NAFLD at risk of liver fibrosis.

## Materials and Methods

### Study population

The adult participants of this study were those who visited the Health Promotion Center of the First Affiliated Hospital of Nanjing Medical University for a health check-up from May 2017 to November 2019. A total of 4880 participants were enrolled. Participants were excluded if they consumed alcohol (i.e., males with > 140 g/week or females > 70 g/week); were carrying or infected by viral hepatitis or other chronic hepatic diseases (e.g., autoimmune liver disease, drug-induced liver disease); were concurrently using lipid-lowering or glucose-lowering medication; had hyperthyroidism or kidney disorders; or had the presence of other dysfunctions of lipid and glucose metabolism. After excluding subjects with the above criteria (Supplement Figure 1), 65 individuals using lipid-lowering medication, 7 individuals using glucose-lowering medication, 13 individuals infected by viral hepatitis, 3 individuals infected by autoimmune liver disease, 2 individuals with sever renal insufficiency and 6 individuals with hyperthyroidism were excluded. 4784 participants were finally included in the study and were further divided into the NAFLD group (*n* = 2902) and the non-NAFLD group (*n* = 1882) according to the results of liver ultrasonography. This study was approved by the Human Research Ethics Committee of the above mentioned hospital (2019-SR-478). The informed consent requirement was exempted because of the retrospective study.

### Data collection

Demographic and vital characteristics of all participants, including age, sex, weight, height, vital signs, medical history, and smoking and drinking status, were collected by a single internist of the health promotion center. Fasting venous blood samples were collected. Glucose, uric acid values, and lipid profiles were assessed with the Chemistry Analyzer AU5800 (Olympus Corporation, Tokyo, Japan). Glycosylated hemoglobin A1c (HbA1c) was determined by high-performance liquid chromatography.

This study retrospectively analyzed data from all participants and found that only 302 participants had plasma insulin concentrations determined by chemiluminescence-based assay. HOMA-IR was assessed as fasting blood glucose (mmol/L) × fasting insulin (uIU/ml)/22.5. The calculation formula of the TyG index was based on previous studies [14, 15].

### Liver fibrosis and severity of steatosis

Transient elastography was performed using the FibroTouch FT100 (Wuxi Hisky Medical Tech. Co., Ltd., Xinwu, Wuxi, China). Liver fibrosis was evaluated using the liver stiffness measurement (LSM) and severity of hepatic steatosis using the fat attenuation parameter (FAP). Ten successful measurements were required for the LSM and FAP to be considered reliable. For these measurements, the IQR/median and success rates must have been < 30% and > 60%, respectively [21]. Patients with NAFLD were grouped by severity according to the FA*P* value as follows: mild, 240 dB/m ≤ FAP < 265 dB/m; moderate, 265 dB/m ≤ FAP < 295 dB/m; and severe, FAP ≥ 295 dB/m. Patients with NAFLD were grouped according to the presence of fibrosis based on LSM values as follows: LSM > 7.3 kPa as liver fibrosis, and LSM ≤ 7.3 kPa as non-liver fibrosis.

### Statistical analysis

Continuous variables were expressed as the mean with standard deviation. Student’s *t* test or one-way ANOVA with Bonferroni correction for pairwise comparisons was used to compare means between two groups or among three groups. Categorical data were expressed as counts and percentages, and comparisons among groups were performed with the Pearson’s chi-squared test. The association of the TyG index with NAFLD and liver fibrosis was estimated by binary or multinomial logistic regression analysis after adjusting for confounding variables, including BMI, HbA1c ALT, AST and GGT. The predictive power of the TyG index for NAFLD and liver fibrosis was estimated with the area under the receiver operating characteristic (AUROC) curve. SPSS Statistics for Windows (version 18.0; IBM Corp., Armonk, N.Y., USA) was used to perform all data analyses, with *P* < 0.05 (two-sided) representing statistical significance.

## Results

### Baseline characteristics of the study population

A total of 4784 participants (3231 males and 1553 females) were finally enrolled, with 2902 (60.7%) confirmed as having NAFLD upon ultrasound. Clinical and laboratory characteristics, grouped by NAFLD status, are listed in Table 1. Compared with the non-NAFLD group, the proportion of men in the NAFLD group was higher. Participants with NAFLD had a positive cardiometabolic risk (i.e., higher BMI, systolic blood pressure (SBP), diastolic blood pressure (DBP), fasting blood glucose (FBG), HbA1c, total cholesterol (TC), triglyceride (TG), low-density lipoprotein cholesterol (LDL-C), and uric acid and lower high-density lipoprotein cholesterol (HDL-C)). Additionally, the TyG index, alanine aminotransferase (ALT), aspartate transaminase (AST) and gamma-glutamyl transpeptidase (GGT) values were higher in participants with NAFLD.

**Table 1:** Baseline characteristics of individuals with or without NAFLD

	Non-NAFLD (*n* = 1882)	NAFLD (*n* = 2902)	*P* value
Age(years)	48.77 ± 10.65	49.15 ± 10.23	0.21
Sex(male/Female)	949/933	2282/620	< 0.01
BMI (kg/m2)	22.85 ± 2.21	26.96 ± 2.94	< 0.01
SBP (mmHg)	122.84 ± 16.94	130.91 ± 16.05	< 0.01
DBP (mmHg)	74.93 ± 10.99	81.31 ± 10.83	< 0.01
FBG (mmol/L)	5.26 ± 1.01	5.75 ± 1.35	< 0.01
HbA1c (%)	5.52 ± 0.63	5.78 ± 0.84	< 0.01
TC (mmol/L)	5.25 ± 1.05	5.41 ± 1.06	< 0.01
TG (mmol/L)	1.34 ± 0.81	2.18 ± 1.47	< 0.01
LDL-C(mmol/L)	3.22 ± 0.76	3.43 ± 0.77	< 0.01
HDL-C(mmol/L)	1.42 ± 0.31	1.21 ± 0.25	< 0.01
TyG index	8.51 ± 0.49	9.04 ± 0.58	< 0.01
Uric acid (umol/l)	311.86 ± 80.11	375.63 ± 84.24	< 0.01
ALT (U/L)	19.74 ± 10.79	31.46 ± 19.99	< 0.01
AST (U/L)	22.22 ± 7.24	25.95 ± 11.99	< 0.01
GGT (U/L)	26.50 ± 22.25	44.77 ± 33.23	< 0.01

Values are presented as mean ± standard deviation

*BMI* body mass index, *SBP* systolic blood pressure, *DBP* diastolic blood pressure, *FBG* fasting blood glucose, *TC* total cholesterol, *TG* triglyceride, *LDL-C* low-density lipoprotein cholesterol, *HDL-C* high-density lipoprotein cholesterol, *TyG* triglyceride and glucose index, *ALT* alanine aminotransferase, *AST* aspartate transaminase, *GGT* gamma-glutamyl transpeptidase

### The TyG index was associated with hepatic steatosis

**Fig. 1:**
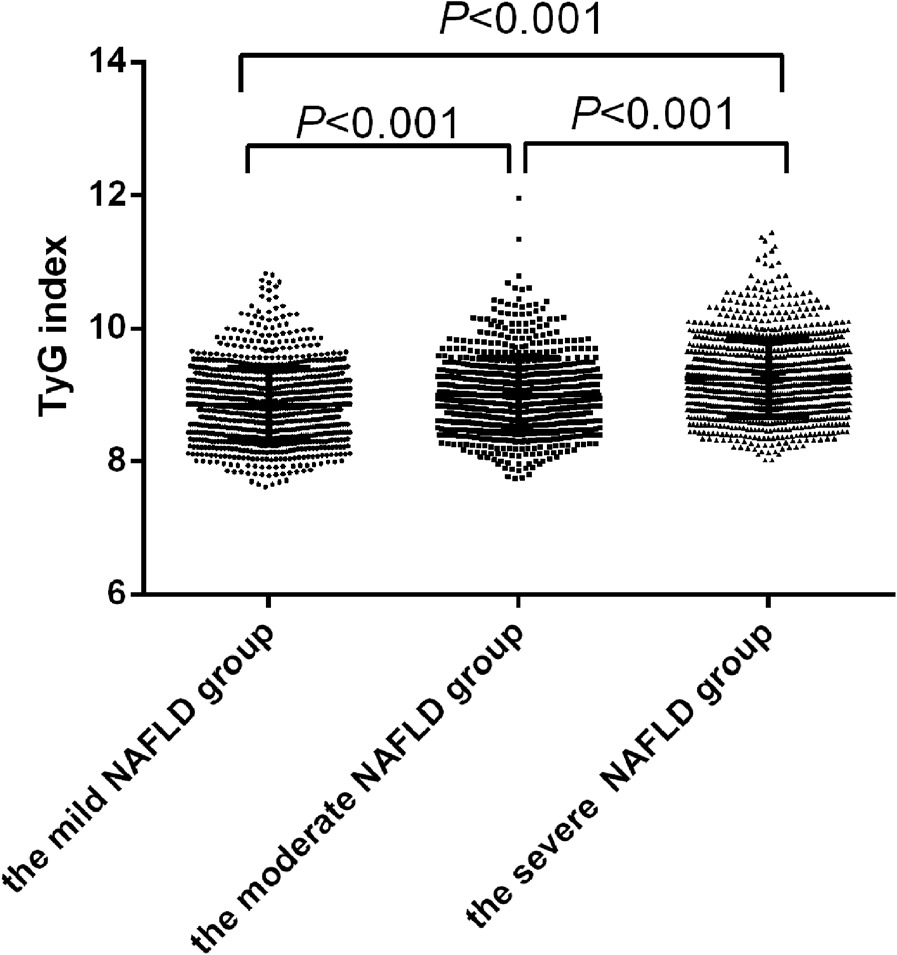
The TyG index in subjects by severity of hepatic steatosis of NAFLD

Participants in the NAFLD group were further divided into three groups according to the FAP value: the mild NAFLD group (*n* = 1043), the moderate group (*n* = 799) and the severe NAFLD group (*n* = 1060). Figure 1 indicated that the TyG index was significantly higher with increased severity of hepatic steatosis (8.85 ± 0.54 vs 8.99 ± 0.55 vs 9.25 ± 0.58, *P* < 0.01).

### Baseline characteristics of NAFLD with and without liver fibrosis

Among participants with NAFLD, 550 had liver fibrosis, and 2352 had non-liver fibrosis. Demographic and clinical features are displayed in Table 2. More participants with liver fibrosis were male and older and had lower HDL-C or elevated BMI, DBP, FBG, HbA1c, SBP, TG, or uric acid than those with non-liver fibrosis (all *P* < 0.01). The TyG index, ALT, AST and GGT were significantly higher in those with liver fibrosis than in those without liver fibrosis.

**Table 2:** Baseline characteristics of individuals with and without liver fibrosis in NAFLD

	Non-liver fibrosis (*n* = 2352)	liver fibrosis (*n* = 550)	*P* value
Age(years)	48.89 ± 10.03	50.27 ± 11.00	< 0.01
Sex(male/Female)	1831/521	451/99	0.03
BMI (kg/m2)	26.58 ± 2.61	28.55 ± 3.65	< 0.01
SBP (mmHg)	129.88 ± 15.67	135.26 ± 16.92	< 0.01
DBP (mmHg)	80.86 ± 10.83	83.25 ± 10.61	< 0.01
FBG (mmol/L)	5.65 ± 1.23	6.18 ± 1.70	< 0.01
HbA1c (%)	5.72 ± 0.78	6.03 ± 1.03	< 0.01
TC (mmol/L)	5.41 ± 1.06	5.45 ± 1.06	0.37
TG (mmol/L)	2.12 ± 1.37	2.45 ± 1.81	< 0.01
LDL-C(mmol/L)	3.43 ± 0.77	3.45 ± 0.76	0.63
HDL-C(mmol/L)	1.22 ± 0.25	1.18 ± 0.26	< 0.01
TyG index	9.00 ± 0.56	9.20 ± 0.64	< 0.01
Uric acid (umol/l)	372.61 ± 84.49	388.56 ± 82.01	< 0.01
ALT (U/L)	29.58 ± 17.92	39.51 ± 25.61	< 0.01
AST (U/L)	24.90 ± 10.41	30.47 ± 16.45	< 0.01
GGT (U/L)	42.92 ± 31.63	52.70 ± 38.39	< 0.01

Values are presented as mean ± standard deviation

*BMI* body mass index, *SBP* systolic blood pressure, *DBP* diastolic blood pressure, *FBG* fasting blood glucose, *TC* total cholesterol, *TG* triacylglyceride, *LDL-C* high-density lipoprotein cholesterol, *HDL-C* high-density lipoprotein cholesterol, *TyG* triglyceride and glucose index, *ALT* alanine aminotransferase, *AST* aspartate transaminase, *GGT* gamma-glutamyl transpeptidase

### The TyG index is associated with hepatic steatosis severity and liver fibrosis

Multinomial logistic regression was used to explore the relationship between the TyG index and the severity of hepatic steatosis, and the group without NAFLD was used as a reference. After adjustment for sex, SBP, DBP, HbA1c, BMI, uric acid, ALT, AST, GGT, and lipid profile including HDL-C, TC, and LDL-C, the TyG index was found to be linked with the severity of hepatic steatosis. The odds ratios (ORs) of the TyG index for mild NAFLD, moderate NAFLD and severe NAFLD were 2.150 (95% CI 1.673–2.764, *P* < 0.001), 2.633 (95% CI 1.977–3.506, *P* < 0.001) and 4.652 (95% CI 3.418–6.311, *P* < 0.001), respectively.

Binary logistic regression was used to explore the association of the TyG index with liver fibrosis. The TyG index was also associated with liver fibrosis upon univariate analysis (OR = 1.764, 95% CI 1.511–2.059, *P* < 0.001). Moreover, this relationship remained significant (OR = 1.313, 95% CI 1.085–1.590, *P =* 0.005), even after adjusting for BMI, sex, age, SBP, DBP, uric acid, HbA1c, ALT, AST, GGT and HDL-C.

### The incidence of NAFLD and liver fibrosis compared across the quartiles of the TyG index

**Fig. 2:**
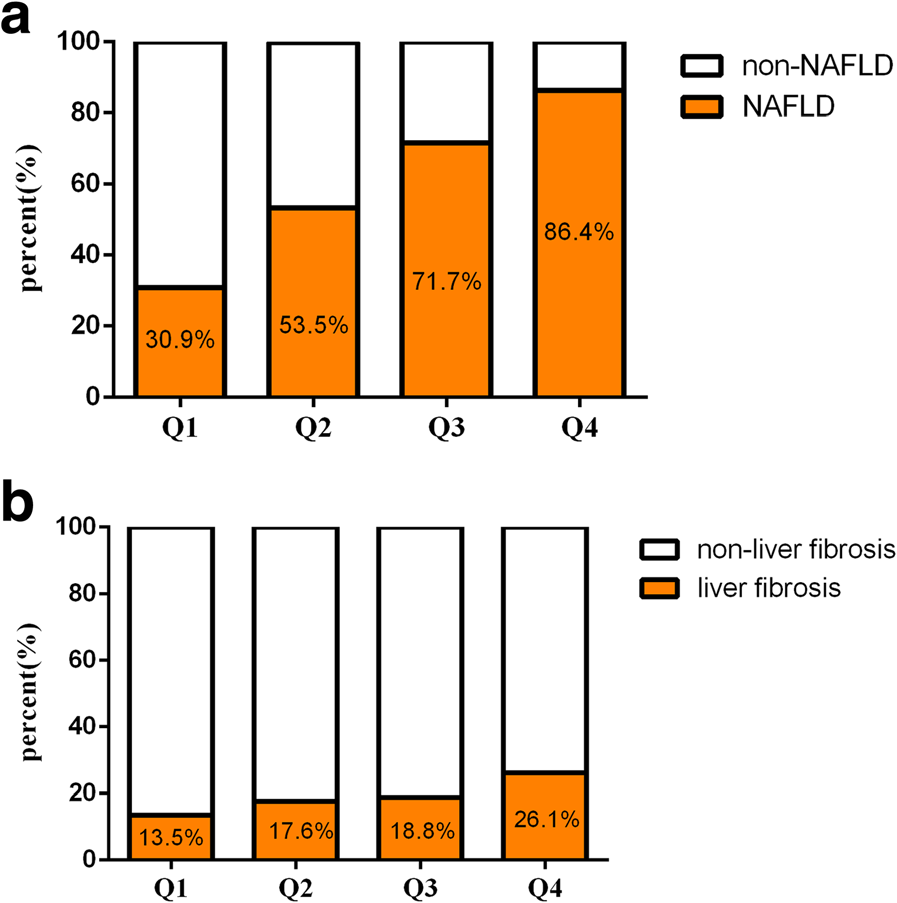
The prevalence of NAFLD and liver fibrosis the by quartiles of the TyG index

All participants were further divided into four groups according to the quartiles of the TyG index. The proportions of NAFLD rose incrementally from the low TyG index value group to the high TyG index value group (Q1 30.9%, Q2 53.3%, Q3 71.7%, and Q4 86.4%, *P* < 0.001) (Fig. 2a). In the subgroup of the NAFLD group, all patients were further divided into four groups according to the quartiles of the TyG index. The proportions of participants with liver fibrosis increased significantly with each TyG index quartile (Q1 13.5%, Q2 17.6%, Q3 18.8%, and Q4 26.1%, *P* < 0.001) (Fig. 2b).

### The TyG index as a predictor for NAFLD and liver fibrosis

**Fig. 3:**
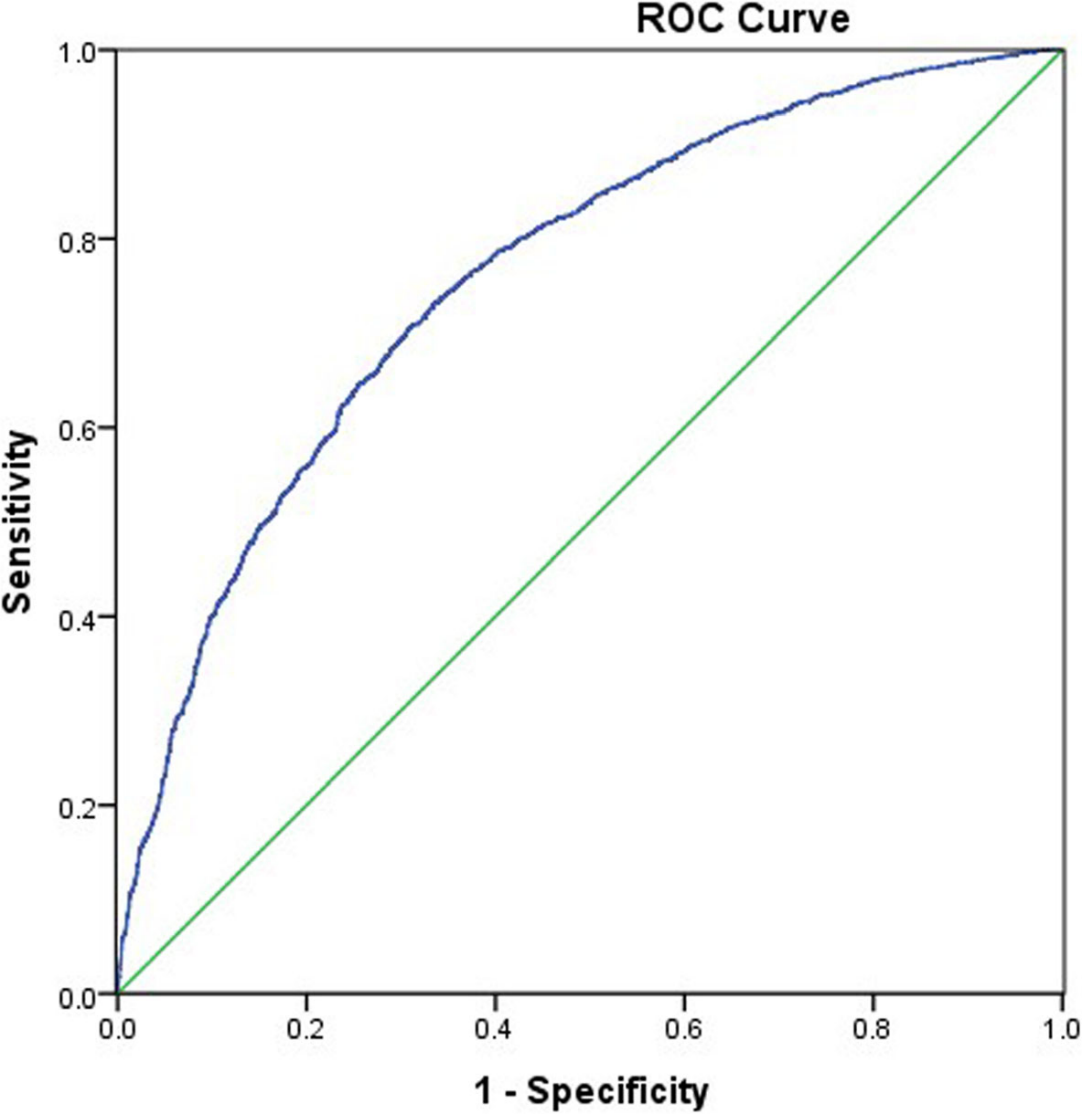
Area under the receiver operating characteristics curve (AUROCs) of the TyG index for NAFLD

**Fig. 4:**
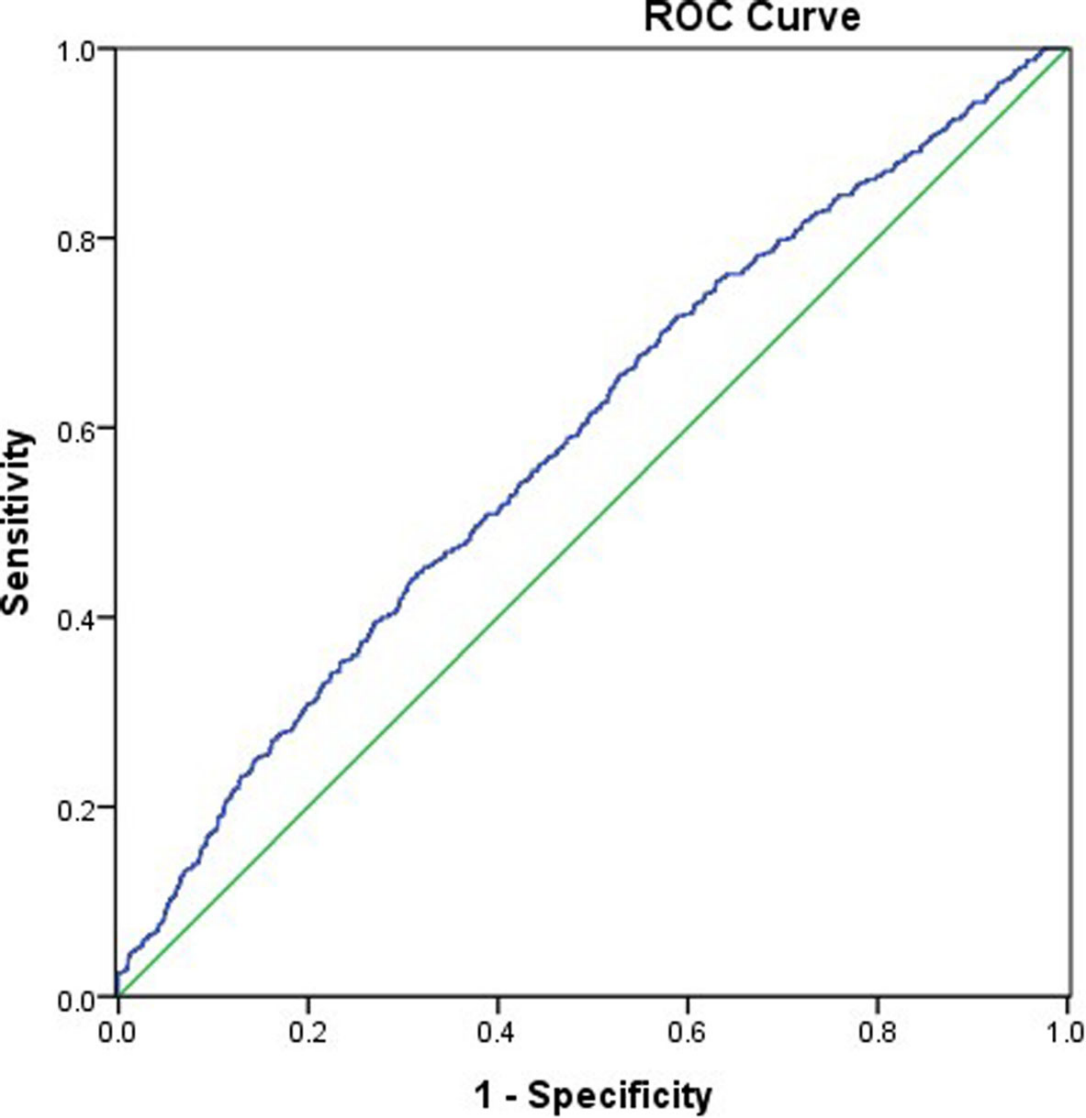
Area under the receiver operating characteristics curve (AUROC) of the TyG index for liver fibrosis

The AUROC value of the TyG index for predicting NAFLD was 0.761 (0.747–0.774, *P* < 0.01), and the cut-off value of the TyG index was 8.70, with 70.6% sensitivity and 69.1% specificity (Fig. 3). That is, the TyG index would be an acceptable predictor of NAFLD if the value was 8.70 or above. The AUROC value of the TyG index for liver fibrosis was relatively smaller than that for NAFLD (0.589, 95% CI 0.562–0.651) (Fig. 4). In this case, the TyG index may not be reliable as a predictor for liver fibrosis in patients with NAFLD.

### Logistic regression analysis of the association of insulin resistance markers with NAFLD and liver fibrosis

Plasma insulin levels were measured in 302 participants, of which 205 had NAFLD and 97 were non-NAFLD. We analyzed and compared the associations of two insulin resistance markers with NAFLD. Participants with NAFLD had a higher TyG index (9.17 ± 0.63 versus 8.53 ± 0.52, *P* < 0.01) and HOMA-IR (5.60 ± 7.04 versus 2.25 ± 2.29, *P* < 0.01) than those with non-NAFLD*.* The univariate analysis showed that both the TyG index (OR = 7.252, 95% CI 4.206–12.503, *P* < 0.001) and HOMA-IR (OR = 1.685, 95% CI 1.382–2.056, *P* < 0.001) were correlated with NAFLD. After adjustment for confounding factors, including sex, DBP, SBP, BMI, TC, LDL-C, HDL-C, HbA1c and uric acid, the relationship between the TyG index and NAFLD remained significant (OR = 3.091, 95% CI 1.597–5.983, *P* < 0.001). Interestingly, after adjusting for HOMA-IR, the association of the TyG index with NAFLD still existed. However, after adjustment for BMI, sex, DBP, SBP, LDL-C, TC, TG, HDL-C, HbA1c and uric acid, there was no longer an association between HOMA-IR and NAFLD.

There were 47 participants with NAFLD who had liver fibrosis and 158 participants without fibrosis. The TyG index (9.52 ± 0.57 vs 9.07 ± 0.60, *P* < 0.01) and HOMA-IR (8.51 ± 9.61 vs 4.73 ± 5.84, *P* < 0.01) were higher in participants with liver fibrosis. The TyG index was still an independent risk factor for liver fibrosis (OR = 3.839, 95% CI 2.024–7.282, *P* < 0.001) after adjusting for confounders including SBP, DBP, sex, age, BMI, HbA1c, uric acid and HDL-C. Interestingly, after adjusting for HOMA-IR, the correlation between the TyG index and liver fibrosis still existed. HOMA-IR (OR = 1.066, 95% CI 1.020–1.114, *P =* 0.005) was related to liver fibrosis in univariate analysis. However, adjusted analysis found no correlation between HOMA-IR and liver fibrosis.

## Discussion

Rapid lifestyle transitions and economic growth in China have contributed to a surprising increase in NAFLD incidence, which reached 29.2% within a short time period [22]. Histologically, NAFLD is subclassified into the specific disorders of non-alcoholic fatty liver (NAFL) and NASH. While NAFL is generally benign, NASH causes cirrhosis, liver cancer, and liver failure, particularly if diabetes is a comorbidity [23, 24]. Patients pay little attention to NAFLD because there are no obvious clinical symptoms. However, an important proportion of individuals with incidentally discovered hepatic steatosis present a high risk of liver fibrosis [25]. Thus, early assessment of individual risk and early intervention for NAFLD is vital for the prevention of liver-related problems and chronic diseases (i.e., type 2 diabetes (T2DM) and CVD).

IR is biologically connected with the development of NAFLD. The gold standard for the measurement of IR is the hyperinsulinemic-euglycemic glucose clamp, but its clinical application is limited because of its time consumption and expense. Alternatively, HOMA-IR, a test based on insulin levels and fasting glucose, most frequently relies on evaluating IR in clinical practice. However, HOMA-IR varies considerably depending on the type of insulin assay and upon which range of fasting plasma insulin levels is considered normal. Insulin levels are usually measured for persons with diabetes mellitus not suitable for the general population. Hence, multiple surrogate markers of IR have recently emerged. The TyG index, as expected, was proven to be correlated with the risk of T2DM. In addition, it is also considered as an effective biomarker to identify NAFLD. Two prospective cohort studies conducted in Chinese and Japanese populations have shown an association between NAFLD incidence and the TyG index [17, 26]. In Korea, Lee SB et al. [20] found that the TyG index could predict NAFLD better than HOMA-IR. To date, there has been little research into the relationship between the TyG index and hepatic steatosis severity and the presence of liver fibrosis in NAFLD. As far as we know, there is only one study including 50 asymptomatic women that showed that the TyG index was the preferred test to screen for simple steatosis and NASH [27]. However, this study did not include men, and the sample size was small. Accounting for confounders, the present study showed an increasing correlation between the TyG index and increasing severity of hepatic steatosis, and likewise, this study found an association with the presence of liver fibrosis. Moreover, this study also validated the ability of the TyG index to identify NAFLD and liver fibrosis. In the present study, the TyG index had a high predictive value for NAFLD, with an AUROC of 0.761. Of note, the optimal TyG cut-off point of 8.70 corresponded with the study results by Zhang S et al., who reported that a TyG index ≥8.5 was effective in screening for NAFLD in a Chinese population [16]. The predictive accuracy of the TyG index for liver fibrosis was limited. The AUROC was 0.589, indicating that the TyG index was not suitable enough to be a predictor for liver fibrosis. Petta S et al. partly corroborated the findings of this study; their study demonstrated that the TyG index had an independent association with liver steatosis. However, in genotype 1 hepatitis C patients, the index was not associated with liver fibrosis [28].

HOMA-IR is another well-known method for estimating IR. In Brazil, Vasques et al. reported better performance using the TyG index than HOMA-IR to estimate IR in Brazilian subjects [29]. Compared with HOMA-IR, several studies indicated that the TyG index was also superior at predicting carotid atherosclerosis, arterial stiffness, diabetes, and hypogonadism [15, 30–32]. Similarly, this study found that the TyG index was more closely tied to NAFLD and liver steatosis than HOMA-IR. The reason for this may be explained that glucotoxicity and lipotoxicity play crucial roles in the regulation of IR, an important pathology connected with NAFLD. The molecular mechanisms remain unclear and still require further study.

### Study strength and limitations

In this study, the TyG index was linked to increasing severity of hepatic steatosis and the presence of liver fibrosis in participants with NAFLD. This is the first study to report these results in a large Chinese population. Another major finding of this study was that the TyG index was an effective marker for identifying individuals with NAFLD but not an acceptable marker for the identification of liver fibrosis. Furthermore, the results of this study indicated that the TyG index was significantly and more closely related to NAFLD and liver fibrosis compared to HOMA-IR after adjustment for confounding factors. However, this study has several limitations. First, this study cannot determine whether the TyG index has a cause-effect relationship with hepatic steatosis or liver fibrosis in NAFLD based on this cross-sectional study. Second, the diagnostic gold standard of liver biopsy was infeasible in this study because of its invasiveness and impracticality in a sample of thousands. Alternatively, ultrasonography and transient elastography were used as noninvasive diagnostic methods in the present study. Finally, data on nutritional and exercise habits were not collected. These factors may have had an effect on circulating TG levels.

## Conclusion

The findings from the present study show that the TyG index is an independent risk factor for hepatic steatosis and the presence of liver fibrosis in participants with NAFLD. The TyG index is more closely related to NAFLD and liver fibrosis than HOMA-IR. Compared with other IR markers, the TyG index is easy to calculate, and the testing cost is inexpensive. Moreover, it appears to be an effective biomarker for identifying individuals with NAFLD. In clinical practice, it is essential to check an individual’s TyG index. If his or her TyG index is more than 8.7, lifestyle modification or drug therapy is necessary for the prevention of the development of NAFLD and even liver fibrosis. Hence, the findings of this study support the wide use of the TyG index for identification and subsequent management of patients with NAFLD.

## Supplementary information

**Additional file 1: Supplement Figure 1.** Flow chart of the study population.

## Data Availability

Data sharing is not applicable to this article as participants did not consent to this.

## Abbreviations

NAFLD: Non-alcoholic fatty liver disease
NASH: Non-alcoholic steatohepatitis
BMI: Body mass index
SBP: Systolic blood pressure
DBP: Diastolic blood pressure
FBG: Fasting blood glucose
TC: Total cholesterol
TG: Triglyceride
LDL-C: Low-density lipoprotein cholesterol
HDL-C: High-density lipoprotein cholesterol
TyG: Triglyceride and glucose index
ALT: Alanine aminotransferase
AST: Aspartate transaminase
GGT: Gamma-glutamyl transpeptidase
LSM: Liver stiffness measurement
FAP: Fat attenuation parameter
HOMA-IR: Homeostasis model assessment of insulin resistance
IR: Insulin resistance
AUROC: Area under the receiver operating characteristic
