# Supplementary figures and images for: The triglyceride-glucose index is associated with the severity of hepatic steatosis and the presence of liver fibrosis in non-alcoholic fatty liver disease: a cross-sectional study in Chinese adults
Source: Lipids Health Dis. 2020 Oct 7;19:218. doi: 10.1186/s12944-020-01393-6 (PMC7541277; doi:10.1186/s12944-020-01393-6)

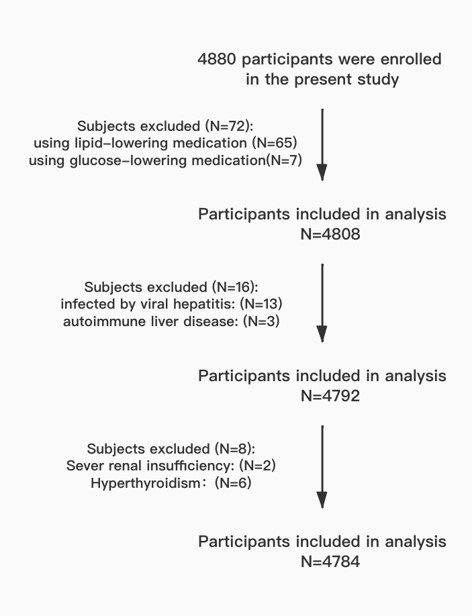

Supplement: Supplementary file 1 — Additional file 1: Supplement Figure 1. Flow chart of the study population. [file 12944_2020_1393_MOESM1_ESM.jpg]
